# Humidity Sensing of Stretchable and Transparent Hydrogel Films for Wireless Respiration Monitoring

**DOI:** 10.1007/s40820-022-00934-1

**Published:** 2022-09-12

**Authors:** Yuning Liang, Qiongling Ding, Hao Wang, Zixuan Wu, Jianye Li, Zhenyi Li, Kai Tao, Xuchun Gui, Jin Wu

**Affiliations:** 1 State Key Laboratory of Optoelectronic Materials and Technologies and the Guangdong Province Key Laboratory of Display Material and Technology, School of Electronics and Information Technology, Sun Yat-Sen University, Guangzhou, 510275 People’s Republic of China; 2 Ministry of Education Key Laboratory of Micro and Nano Systems for Aerospace, Northwestern Polytechnical University, Xi’an, 710072 People’s Republic of China

**Keywords:** Stretchable and transparent humidity sensors, Hydrogel film, Wireless and wearable sensor, Respiration monitoring, Ultrasensitive

## Abstract

Respiratory monitoring plays a pivotal role in health assessment and provides an important application prospect for flexible humidity sensors. However, traditional humidity sensors suffer from a trade-off between deformability, sensitivity, and transparency, and thus the development of high-performance, stretchable, and low-cost humidity sensors is urgently needed as wearable electronics. Here, ultrasensitive, highly deformable, and transparent humidity sensors are fabricated based on cost-effective polyacrylamide-based double network hydrogels. Concomitantly, a general method for preparing hydrogel films with controllable thickness is proposed to boost the sensitivity of hydrogel-based sensors due to the extensively increased specific surface area, which can be applied to different polymer networks and facilitate the development of flexible integrated electronics. In addition, sustainable tapioca rich in hydrophilic polar groups is introduced for the first time as a second cross-linked network, exhibiting excellent water adsorption capacity. Through the synergistic optimization of structure and composition, the obtained hydrogel film exhibits an ultrahigh sensitivity of 13,462.1%/%RH, which is unprecedented. Moreover, the hydrogel film-based sensor exhibits excellent repeatability and the ability to work normally under stretching with even enhanced sensitivity. As a proof of concept, we integrate the stretchable sensor with a specially designed wireless circuit and mask to fabricate a wireless respiratory interruption detection system with Bluetooth transmission, enabling real-time monitoring of human health status. This work provides a general strategy to construct high-performance, stretchable, and miniaturized hydrogel-based sensors as next-generation wearable devices for real-time monitoring of various physiological signals.

**Supplementary Information:**

The online version contains supplementary material available at 10.1007/s40820-022-00934-1.

## Highlights

- A general strategy of fabricating thickness-controllable hydrogel films with diverse polymer networks for humidity sensing is proposed. The hydrogel film humidity sensor displays an unprecedented sensitivity up to 13,462.1%/%RH.
- The stretchable humidity sensor can work normally under large tensile strains with even enhanced sensitivity, which makes the humidity responding property of hydrogel mechanically programmable.
- A wireless respiratory interruption detection mask with Bluetooth transmission function is developed based on the sensor, enabling the real-time, wireless and accurate acquisition of user's breathing state, such as the interval and breathing frequency and apnea.

**Supplementary Information:**

The online version contains supplementary material available at 10.1007/s40820-022-00934-1.

## Graphical abstract

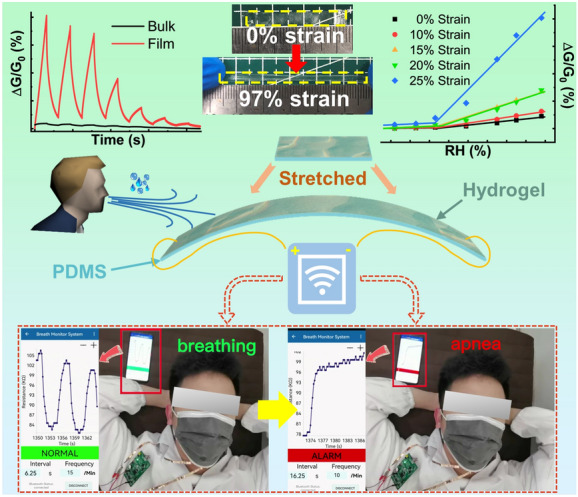

## Introduction

Portable or wearable electronic devices with good shape-conformability and specific stimuli-responsiveness are currently attracting extensive attention and have a widespread employment in soft robots, healthcare, artificial intelligence, and human–machine interaction [1–6]. To better adapt to more application scenarios, various functionalities are expected to be introduced through refined material and structural engineering strategies [7–12]. For instance, transparency is an important prerequisite feature in device visualization, and the next-generation flexible transparent electronics play an essential role in displays, military camouflage, and optometry products [13–17]. Typically, it is anticipated that convenient, effective and real-time respiratory monitoring can be performed using these wearable sensing electronics, since exhaled breath contains a series of variable detectable physiological signals. Wherein, humidity is an obvious indicator that can be used for respiration monitoring, and high-performance humidity sensors can effectively distinguish different respiratory patterns and play an important role in health assessment and disease prediction [18–20]. For example, patients with sleep apnea syndrome (SAS) are prone to apnea, and patients with pneumonia, bronchitis, and asthma are often accompanied by symptoms of tachypnea, so real-time monitoring of respiratory rate is beneficial for their adjuvant therapy [21, 22]. In general, a high-performance humidity sensor needs to meet the requirements of high sensitivity, fast response and recovery speeds, wide monitoring range, good stability, low fabrication cost and simplicity, and it can also be exploited to achieve non-contact human–machine identification and even perceive plant growth by detecting subtle changes in relative humidity (RH) caused by leaf transpiration, showing broad application prospects [23, 24].

Generally, traditional flexible humidity sensors are constructed by integrating non-stretchable humidity sensing materials, such as graphene oxide (GO), reduced graphene oxide (rGO), single-walled carbon nanotube (SWCNT), micro/nanostructured metal oxides, conductive polymer, etc., onto flexible substrates, which always bear limited deformability and struggle to meet the requirements of wearable devices [25–29]. For instance, Cai and coworkers fabricated a flexible humidity sensor composed of an interdigitated rGO/GO/rGO structure on polyethylene terephthalate (PET) substrate fabricated by laser direct writing method, which exhibited obvious response to a wide range of RH (6.3%-100%) as well as the long-term stability [30]. Subsequently, Ma et al. developed a flexible substrate-free yarn-shaped humidity sensor with excellent sensing capabilities, enabling wireless respiration monitoring [31]. Although remarkable humidity responsiveness is achieved, these humidity sensors are prone to permanent failure after stretching and have poor deformation resistance. Interestingly, Zhou and colleagues reported a wearable textile-based humidity sensor based on SWCNT/Poly(vinyl alcohol) (PVA) filaments fabricated via a wet-spinning process [32]. As a result, this sensor was highly stretchable but had a limited humidity monitoring range (60–100%) and inferior response. Overall, it is still challenging to develop a humidity sensor with ultra-responsiveness, wide monitoring range, high stretchability and transparency simultaneously [33, 34].

Recently, hydrogels consisting of three-dimensional networks and numerous water molecules have emerged as a promising stretchable material for the fabrication of wearable electronics, thanks to its intrinsic stretchability and ionic conductivity [35–41]. Attractively, the ionic conductivity of hydrogels is directly related to their water content, and the adsorption and desorption of water molecules will inevitably cause its variation. Based on this characteristic, there are few flexible and stretchable hydrogel-based humidity sensor have been fabricated, but with suboptimal sensitivity and bulky structure [42–45]. In order to further improve the performance of hydrogel-based humidity sensors and promote the practical applications, several significant challenges need to be tackled. First, the responsiveness of hydrogel-based sensors to humidity needs to be greatly improved. Considering that the bulk hydrogels are currently used as sensing materials, the diffusion of water molecules in the bulk phase is inefficient and time-consuming, and therefore increasing their specific surface area is expected to achieve explosive growth in humidity-sensing performance due to the exposure of more adsorption sites. Second, hydrogels always have poor stability due to the tendency of water evaporation and freezing, and the improvement of their anti-freezing and water retention abilities is beneficial for the environmental stability and broad application scope of devices. Then, the current humidity sensor as wearable electronics should also develop in the direction of miniaturization, light weight, low cost, stability and processability, just like the current integrated circuit industry to achieve actual commercial application. This further requires the development of a general mass-producible technique to fabricate microscale hydrogels, given that current research effort is mainly devoted to the functional development of bulk hydrogels and further miniaturization studies are rarely involved. Finally, in addition to exploring humidity sensing device itself, the development of smart humidity sensing systems incorporating specially designed circuits is also necessary for practical wearable applications, such as the real-time respiration monitoring. Unfortunately, wireless, wearable, and high-sensitivity hydrogel-based humidity sensing systems for sleep apnea monitoring have not been reported so far.

**Fig. 1:**
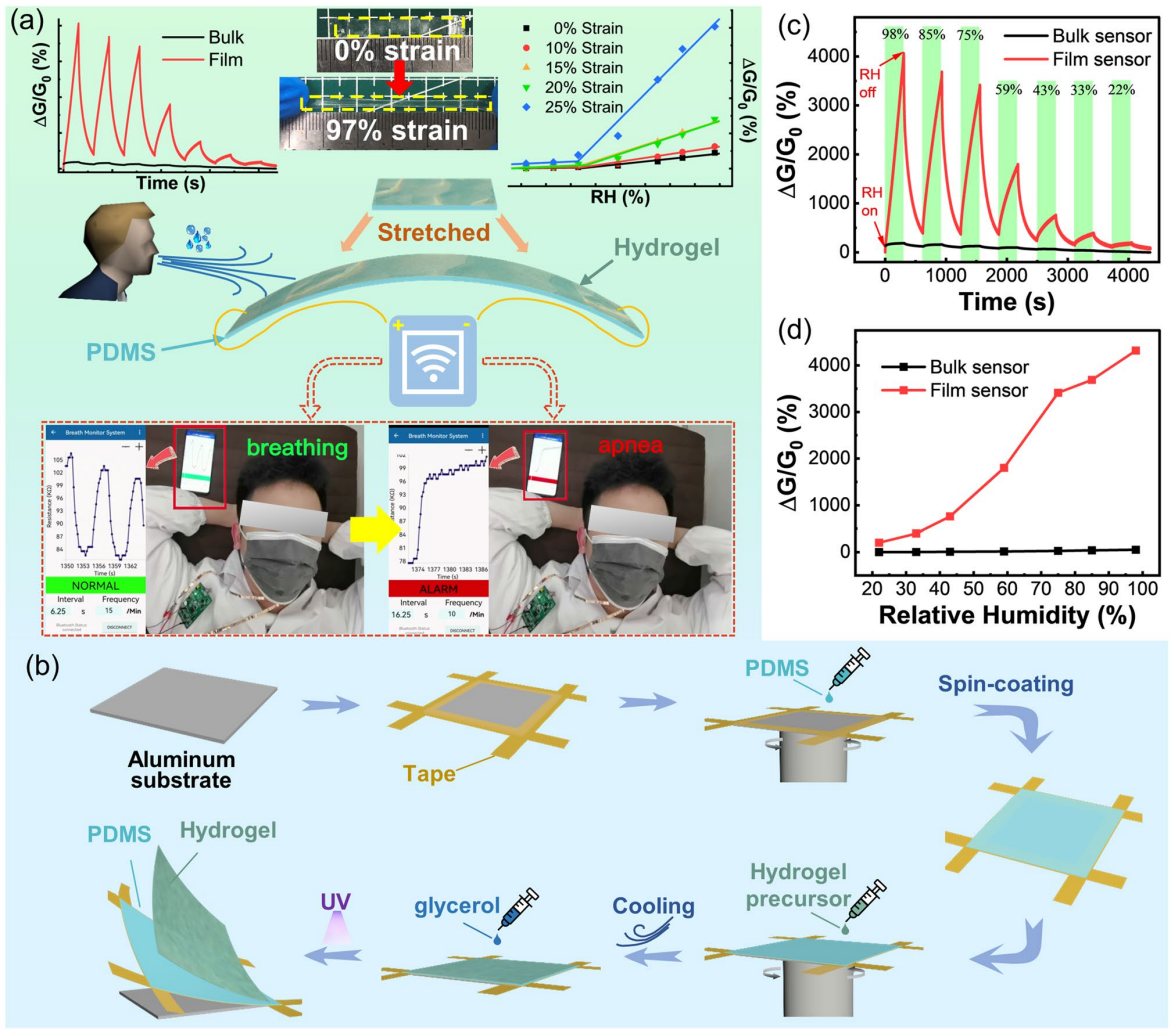
**a** Schematic and photographs show the excellent performance of the stretchable hydrogel film humidity sensor and its practical wearable application for wireless respiration monitoring. **b** Scheme illustrating the preparation process of hydrogel film with the polydimethylsiloxane (PDMS) film substrate. **c** Time-dependent responses of the bulk and film hydrogel sensors to different RH. The green and white areas indicate that the sensor is in high and low (11%) RH environments, respectively. **d** The relationship between RH and the responses of bulk and film sensors

In this work, ultra-high-performance, stretchable, transparent, and cost-effective humidity sensors and corresponding wireless sensing system are developed, enabling real-time respiration monitoring even under stretching (Fig. 1a). Importantly, we propose a general method for preparing hydrogel films with a controllable thickness (Fig. 1b). The hydrogel film refers to a hydrogel with a thickness of micrometers (usually below 100 μm), which is several orders of magnitude smaller than that of bulk hydrogel (about 1 cm). The method is extremely beneficial for the enhanced adsorption (at high RH) and desorption (at low RH) of water molecules and the integration of flexible electronics in the future, and the humidity sensor based on the obtained hydrogel films exhibits greatly improved response compared with bulk hydrogels (Fig. 1c-d). In particular, hydrogel films containing different polymer networks have been successfully prepared successively, which demonstrates the versatility of our attempted thin-filmization method in boosting the humidity responsiveness of hydrogels. Furthermore, through the regulation of the introduced components, the sensitivity of the sensor has been further improved to 13,462.1%/%RH, superior to that of the current hydrogel-based humidity sensor and even the state-of-the-art humidity sensor. To improve the stability of the hydrogel, polyols or hygroscopic salt (LiBr) are introduced, resulting in good stability and reliability of the sensor. Concretely, the effects of film thickness and salt concentration on the humidity sensing performance were systematically studied, and the sensing mechanism was also elucidated using the electrochemical impedance spectroscopy (EIS). This work provides ideas for using hydrogel films to prepare deformable humidity sensors with higher performance and more application scenarios.

## Experimental Section

### Synthesis of the Bulk and Film Hydrogel

#### Synthesis of the PAM/Carrageenan Bulk Hydrogel

Chemicals including acrylamide (AM), N,N-methylene bisacrylamide (MBA), and potassium chloride (KCl) were purchased from Sigma-Aldrich. Carrageenan and irgacure2959 (photoinitiator) were purchased from Aladdin. The PAM/Carrageenan hydrogel was prepared via a one-pot sol–gel process. Firstly, 7.5 g AM, 1.5 g carrageenan, 0.005 g MBA, 0.09 g KCl, 0.1 g photoinitiator, and 41 mL deionized water were added to a flask. To disperse the chemicals uniformly, the solution was stirred at 95 °C for 5 h at 550 rpm min^−1^. A rubber stopper was deployed to seal the mouth of the flask to prevent moisture from evaporation. After heating and stirring, a flowable PAM/Carrageenan hydrogel precursor solution was obtained, which was poured into a glass petri dish, and then placed in a refrigerator at 6 °C for 1 h. After refrigeration, the first network of carrageenan was formed. The samples were then irradiated with UV light for 2 h. Under the combined action of MBA, photoinitiator, and UV light, AM was induced to polymerize to form the second network of PAM, leading to the formation of PAM/Carrageenan DN hydrogel. A piece of hydrogel with the size of 2 × 1 × 1 cm^3^ hydrogel was then soaked in glycerol solution with controlled concentrations for 2 h.

#### Preparation of the PAM/Carrageenan Hydrogel Film

The preparation process of the PAM/Carrageenan hydrogel precursor solution is the same as above. Dowsil 184 silicone elastomer base and curing agent were poured into a plastic cup at a ratio of 10:1 and mixed for 60 s. High-temperature resistant tapes were stuck around several 3 × 3 cm^2^ aluminum (Al) sheets to facilitate tearing off the film hydrogel after synthesis (Fig. 1b). 2 g of the PDMS mixture was spin-coated on the Al sheet at 1000 rpm for 30 s. Subsequently, the Al sheet was placed on a heating plate with a temperature of 80 °C for PDMS curing. After 2 h, the PDMS surface was treated by oxygen plasma (120 W, 5 min) to render it hydrophilic by placing the sample in a vacuum plasma cleaner (PT-2S, POTENTLUBE P). About 5 mL of PAM/Carrageenan hydrogel precursor solution was taken with a plastic tip dropper and then spin-coated on the Al sheet carrying the PDMS substrate at different speeds for 30 s to obtain hydrogel layers with different thicknesses. After spin-coating, the samples were placed in a refrigerator at 6 °C for 1 h to form the first network of carrageenan under the action of potassium ions. Then 2 mL of glycerol solution was added dropwise to cover the surface of samples. The samples were placed under a UV lamp for photo-initiation for 2 h to polymerize AM for the formation of PAM. After UV initiation, the glycerol solution was poured out, and the residual solution on the surface of the film hydrogel was blotted with filter paper.

#### Synthesis of the PAM/Tapioca Bulk Hydrogel

The PAM/tapioca hydrogel was prepared via a one-pot sol–gel process. Firstly, 7.5 g AM, 1.5 g tapioca, 0.005 g MBA, 0.09 g KCl, 0.1 g photoinitiator, and 41 mL deionized water were added to a flask. To disperse the chemicals uniformly, the solution was stirred at 95 °C for 5 h at 550 rpm min^−1^. A rubber stopper was used to seal the mouth of the flask to prevent the moisture from evaporating. After heating and stirring, a flowable PAM/tapioca hydrogel precursor was obtained, which formed PAM/tapioca bulk hydrogel after being radiated with UV light for 2 h.

#### Synthesis of the PAM/Tapioca Hydrogel Film

The prepared PAM/Tapioca hydrogel precursor solution could also be used to fabricate the PAM/Tapioca hydrogel film. The preparation of PAM/Tapioca hydrogel film is similar to that of PAM/Carrageenan hydrogel film except without using PDMS as the supporting layer. Specifically, after the Al sheet was subjected to oxygen plasma cleaning with a power of 120 W for 5 min, 5 mL of the precursor solution was dropped on it to obtain a hydrogel precursor layer after a spin-coating for 30 s. Then, the precursor layer was converted to PAM/tapioca hydrogel film after being radiated with UV light for 2 h for the cross-linking of AM.

### Material Characterization

The differential scanning calorimetry (DSC) spectra were obtained on a Netzsch DSC-204-F1 at a cooling rate of 5 °C min^−1^ from 25 to – 120 °C. The morphology of hydrogel films was characterized using polarizing optical microscope (Axio Scope A1 pol, Zeiss). The transmittance spectra were acquired using the ultraviolet spectrophotometer (Evolution 220, Thermo Fisher). The Fourier transform infrared (FTIR) spectra were acquired on a Thermo Scientific Nicolet 6700 FT-IR Spectrometer.

### Humidity Sensing Characterization

A homemade static humidity characterization system was deployed for the RH sensing test at room temperature. Taking advantage of the characteristic that the RH of the air above the saturated salt solution is constant under specific temperature and pressure, a sealed glass bottle with a specific RH in the internal air was created using specific salt and further used as the humidity testing chamber. The RH of 11, 22, 33, 43, 59, 75, 85, and 98% were obtained using the saturated solutions of lithium chloride (LiCl), potassium acetate (CH_3_COOK), magnesium chloride (MgCl_2_), potassium carbonate (K_2_CO_3_), sodium bromide (NaBr), sodium chloride (NaCl), KCl, and potassium sulfate (K_2_SO_4_), respectively. During the testing, changing the RH around the sensor was achieved by rapidly transferring the hydrogel sensor from one sealed glass bottle into another bottle with a different RH. Both the conductance and capacitance of the sensor were measured using an LCR meter (Tonghui, TH2832) at 200 Hz. To measure the humidity-sensing properties at different strains, two binders were utilized to fix the stretched hydrogel sensor on a glass slide after applying certain tensile strains such as 10, 15, and 20%.

## Result and Discussion

### Environment Tolerant PAM/Carrageenan Hydrogel for Humidity Detection

PAM hydrogel is an outstanding candidate for the fabrication of flexible wearable electronics due to its excellent stretchability (up to 1500% strain) and ion transport ability [46]. In particular, it contains abundant polar amide groups that can easily form hydrogen bonds with water molecules, showing great potential for humidity monitoring. Herein, PAM/Carrageenan double network (DN) hydrogels were prepared based on the UV polymerization method, where carrageenan is physically cross-linked, leading to better mechanical properties due to the introduction of an additional energy dissipation mechanism. However, severe dehydration always exists due to the abundant free water in hydrogels, which greatly hinders their practical application scenario, humidity detection range and further commercialization [47–49]. Especially, the hydrogel film is hard to form if without introducing humectant in the aqueous precursor solution, since it dehydrates easily and rapidly after spin-coating under the function of UV light. To address this problem, environmentally stable PAM/Carrageenan organohydrogels were obtained by immersing the pre-hydrogels in glycerol solution using a simple solvent replacement method (Fig. 1b). Inherently, polyols like glycerol have excellent hygroscopic ability, because abundant hydroxyl groups on them can bind with water molecules through hydrogen bonds, which can greatly reduce the content of free water in the hydrogels and ultimately enhance their stability [50–53]. By comparing the (FTIR) spectra of the hydrogels with and without immersion in Gly solution, the intensity of the O–H stretching peak at 1039 cm^−1^ of the hydrogels immersed in Gly solution is much higher than that without Gly immersion, indicating that Gly was successfully introduced into the hydrogel by this solvent replacement method (Fig. S1).

**Fig. 2:**
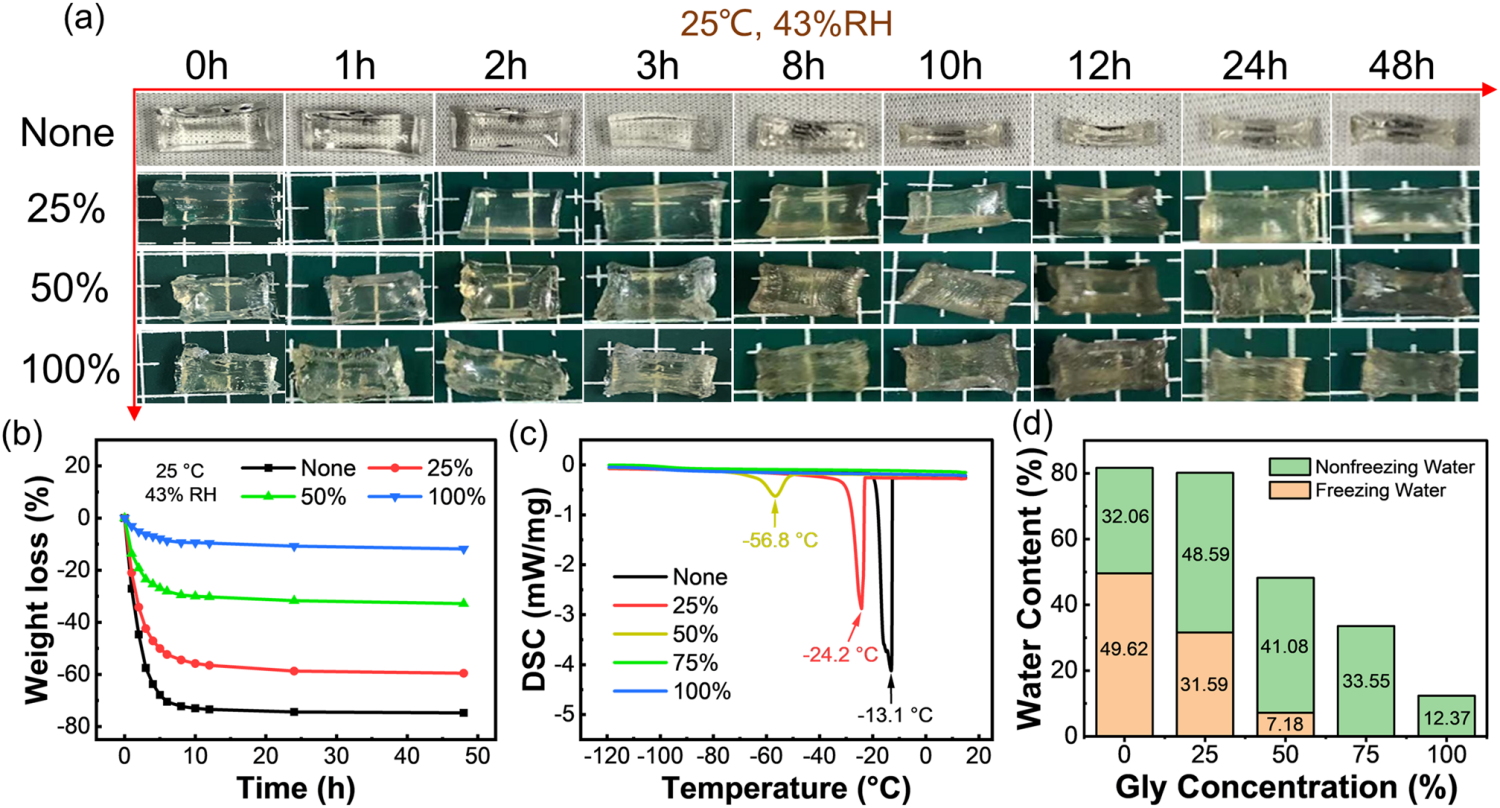
**a** Photographs of the morphology evolutions of the PAM/Carrageenan DN hydrogels that were not soaked and soaked in 25, 50, and 100% glycerol solutions, respectively, after being stored in a dry environment of 25 °C and 43% RH for 48 h. **b** Time evolution of the weight loss percentage of the hydrogels in **a** when stored at 25 °C and 43%RH. **c** DSC curves of hydrogels that were not soaked in glycerol and soaked in 25, 50, 75, and 100% glycerol solutions, respectively. **d** Contents of freezing and nonfreezing water in hydrogels soaked in glycerol solutions with different concentrations

Whereafter, a series of PAM/Carrageenan organohydrogels were prepared by soaking the hydrogel in different mass ratios of Gly aqueous solutions (25%, 50%, and 100%), and their moisturizing ability was compared with that of original hydrogel. Figure 2a shows the morphology evolution of the PAM/Carrageenan hydrogel and organohydrogels after being stored in a dry environment of 25 °C and 43% RH for 48 h. Obviously, the hydrogel without Gly immersion dried and shrunk significantly after 3 h, and the mass loss reached as high as 75% after 48 h. Attractively, the organohydrogels with the water/Gly binary solvent did not deform obviously in the dry environment for 48 h, and the weight loss of the organohydrogel soaked in 100% Gly solution was only 11% (Fig. 2b). This manifests that the introduction of Gly can greatly enhance the moisturizing properties of the hydrogels, thereby improving the stability of hydrogel-based devices. Additionally, these Gly-impregnated organohydrogels exhibit remarkable freezing resistance due to the hindered formation of hydrogen bonds between water molecules at sub-zero temperatures. According to the (DSC) spectra (Fig. 2c), the freezing points of these hydrogels gradually decreased from – 24.2 ℃ to below – 120 ℃ with increasing Gly content. Figure 2d quantitatively counts the respective contents of frozen water and non-frozen water in these samples. After the incorporation of glycerol, the water content in the hydrogel decreases correspondingly and is accompanied by an increase in the proportion of nonfreezing water that bonded with Gly molecules by forming hydrogen bonds, thus resulting in an excellent frost resistance [54, 55]. As a result, the solvent replacement strategy is an effective means to improve the stability and applicability of hydrogels in various environments by replacing part of water with organic molecules.

Utilizing this organohydrogel with excellent environmental stability as a humidity-sensing material, a flexible humidity sensor was constructed by attaching two copper electrodes to its two ends. By supplying an alternating current (AC) (0.5 V, 200 Hz) to the sensor, its humidity-sensing properties were tested by monitoring the conductance change when alternately exposing it to 11% RH and a specifically different RH environment. Testing with AC is to exclude the interference of gases in the external environment. If a stable direct current (DC) is used, redox gases, such as oxygen, will electrochemically react at the interface between the organohydrogel and the electrodes. As such, the varied concentration of these gases will interfere with the humidity sensor, while utilizing the AC will not have this problem. Also, different quantitative RH around the sensor can be changed by quickly placing the sensor in a sealed bottle with different saturated salt solutions, since the RH above their liquid level is different and stable, which is determined by the type of salt [56]. As shown in Figs. 1c and S2, the conductance of the hydrogel sensor increases with RH, indicating its humidity monitoring capability. Here, the response of the humidity sensor is defined as *ΔG/G*_*0*_, where *ΔG* is the change in the conductance of the sensor at the target RH compared to the initial conductance (*G*_*0*_) at 11% RH. Figure 1d shows that the higher the RH of the environment where the sensor is located, the higher the response, which can be used for humidity calibration. Fundamentally, this humidity sensing capability is attributed to the fact that different RH environments can change the water content in the hydrogels through the facile adsorption and desorption of water molecules, which in turn change the conductivity (σ) of the ionically conductive hydrogels. Quantitatively, σ can be calculated using the following equation:

1 $$\sigma =\sum_{i}{n}_{i}{\mu }_{i}{Z}_{i}e$$

where ${n}_{i}$ is the carrier concentration, ${\mu }_{i}$ is the ion mobility, ${Z}_{i}$ is the valence state of the mobile ionic charge, and *e* is the unit charge [57]. When exposed to high humidity, the adsorption of water molecules in the hydrogel is significantly stronger than the desorption thanks to the presence of a plethora of unbonded hydrophilic groups such as hydroxyl and amide groups, and hydrogel can adsorb water molecules from the surrounding environment by forming hydrogen bonds until the equilibrium of water molecule adsorption and desorption is reached. The concentration of the Gly solution in which the film hydrogel is soaked has little effect on the humidity responsive behavior of it (Fig. S3). During this process, there are several factors accounting for the increased conductivity of the film hydrogel with the increased content of water molecules. First, the solubility of salts involved in ionic conduction increases, thereby increasing the ${n}_{i}$ and σ. More importantly, the migration ability and mobility of ions enhance with the higher content of water. Furthermore, the hindering effect of polymer network on the transport of ions can be weakened due to the swelling effect of the hydrogel film, thus leading to an increase in ${\mu }_{i}$ and σ. As the humidity decreases, the desorption of water molecules dominates and the conductance eventually falls back to the original state since the hydrogen bonds formed are reversible, reflecting the reversible and repeatable humidity detection capability of the hydrogel. However, the response of conventional bulk hydrogel is rather unsatisfactory due to the limited interaction area between moisture and hydrophilic groups on hydrogel (Fig. S2), and therefore further exploration for general strategies to enhance the humidity sensing performance of hydrogels is urgently needed.

### Universal Miniaturization Approach for Superior Humidity Sensing with Clarified Sensing Mechanism

**Fig. 3:**
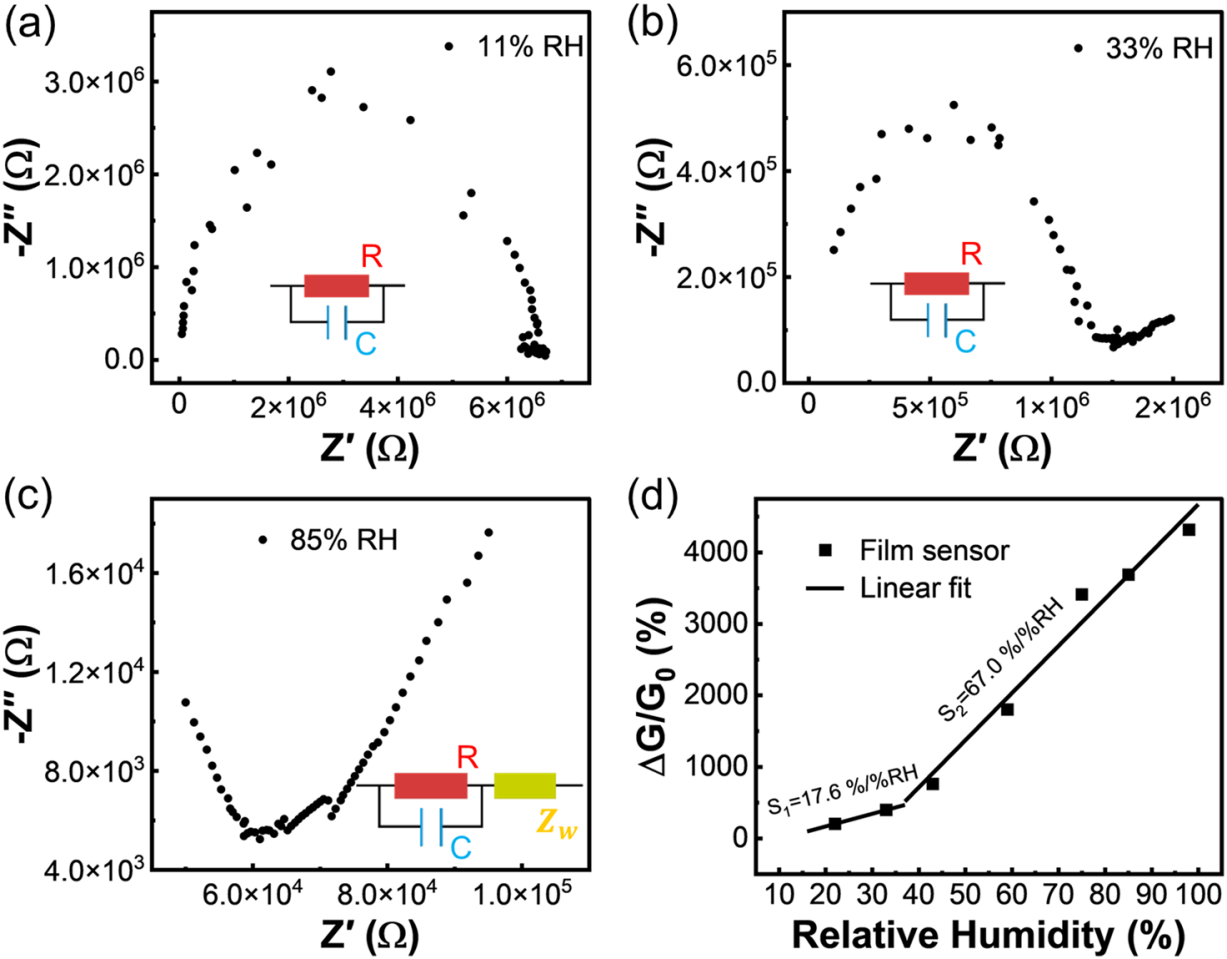
**a-c** The complex impedance spectra of the humidity sensor under 11, 33, and 85% RH environment. **d** Piecewise fitting of response versus RH within 11–98% RH

Given that the humidity sensing performance of hydrogels is directly related to the adsorption efficiency of water molecules, the exposure of more adsorption sites should be an effective means to enhance the response to RH, that is, the development of miniaturized hydrogels with enlarged surface area is quite conducive to the high sensitivity. It not only greatly optimizes the sensing performance of the hydrogel, but also facilitates the miniaturized design of related devices and the realization of the eventual integrated flexible electronics. Whereas, hydrogels have poor processability compared to rigid materials, which results in slow miniaturization progress. To implement this goal, we propose a general method for the preparation of hydrogel films by in-situ polymerization of spin-coated precursor solutions with a certain viscosity on substrates (Fig. 1b). As shown in Fig. 1d, PAM/carrageenan hydrogel film was prepared according to this. As expected, compared with that of the bulk sensor, the response of the hydrogel film-based humidity sensor grew dramatically (Fig. 1c-d). Owing to the thin-filmization process, the specific surface area of the sensing material, that is, the ratio of the surface area to the volume of the hydrogel is greatly increased. The specific surface area of the 54.51-μm-thick film is as high as 0.037 μm^−1^, which is much larger than that of the bulk counterpart (0.0005 μm^−1^) (Fig. S4a). As such, the adsorption and desorption of water molecules by the hydrogel film are more efficient than that of the bulk hydrogel, as the relative weight variation of the hydrogel film is much higher than that of the bulk hydrogel under the same humidity variation (Fig. S5). Furthermore, the hydrogel film-based sensor possesses an extremely low initial conductance under 11% RH due to the desorption of more water molecules in this dry environment, which can also improve its sensitivity to humidity due to the low baseline. Specifically, the initial conductance can be as low as 2.75 × 10^–8^ S when the thicknesses of the film is 54.61 μm, which is four orders of magnitude lower than that of the bulk hydrogel (5 × 10^–4^ S) (Fig. S4a). Moreover, we notice that the response of the hydrogel film-based sensor does not increase linearly with RH. Within 11% to 37% RH, the response grows slowly and has a low sensitivity calculated from the slope of the response versus RH curve. Whereas in the range of 37% to 98% RH, the sensor has greater sensitivity, and there must be two distinct conductance change mechanisms here that enable the piecewise response (Fig. 3).

To further explore the sensing mechanism of hydrogel film-based humidity sensor, its EIS spectra under different RH were tested, and the corresponding equivalent circuit was also analyzed [58]. Under the dry environment (11% RH), the complex impedance spectrum is a complete semi-circle, and the equivalent circuit is represented by the parallel connection of film resistors and capacitors (Fig. 3a). In this case, the ion diffusion effect inside the film is not obvious, only a few water molecules are adsorbed on the surface of the hydrogel film, and the conductivity is mainly caused by the hopping of ions between surface water molecules. At 33% RH, the semi-circle of the spectrum is still complete, but a short-slanted line begins to appear in the low-frequency region (Fig. 3b). It is because the series Warburg impedance starts to be added to the equivalent circuit, but its effect on the shape of the spectrum is not yet obvious, and the resistance changes little, resulting in an insignificant change in the conductance of the hydrogel. As the humidity further increases (37%-98% RH), the semi-circle of the complex impedance spectrum tends to disappear gradually, and the oblique line becomes manifestly longer (Fig. 3c). The series Warburg impedance becomes the main factor affecting the shape of the spectrum, and the resistance decreased rapidly, leading to a fast upward trend of the conductance of the hydrogel. Within this range, more water molecules are adsorbed on the surface and diffuse into the interior to form a continuous hydration layer, allowing direct diffusion of ions. As a result, the response versus RH curve shows two linear regions. For better distinction, the sensitivity in the low humidity range is described by S_1_, while that in the relatively high humidity range is defined as S_2_. By performing a proper linear fit to the response versus RH curve (Fig. 3d), S_1_ and S_2_ of the hydrogel film-based humidity sensor were estimated to be 17.6 and 67.0%/%RH, respectively, which are two orders of magnitude higher than that of the bulk counterpart (0.2%/%RH). As demonstrated above, the thin-filmization of the hydrogels can greatly improve their sensitivity to humidity, demonstrating its feasibility of optimizing the humidity sensing performance by structural engineering.

**Fig. 4:**
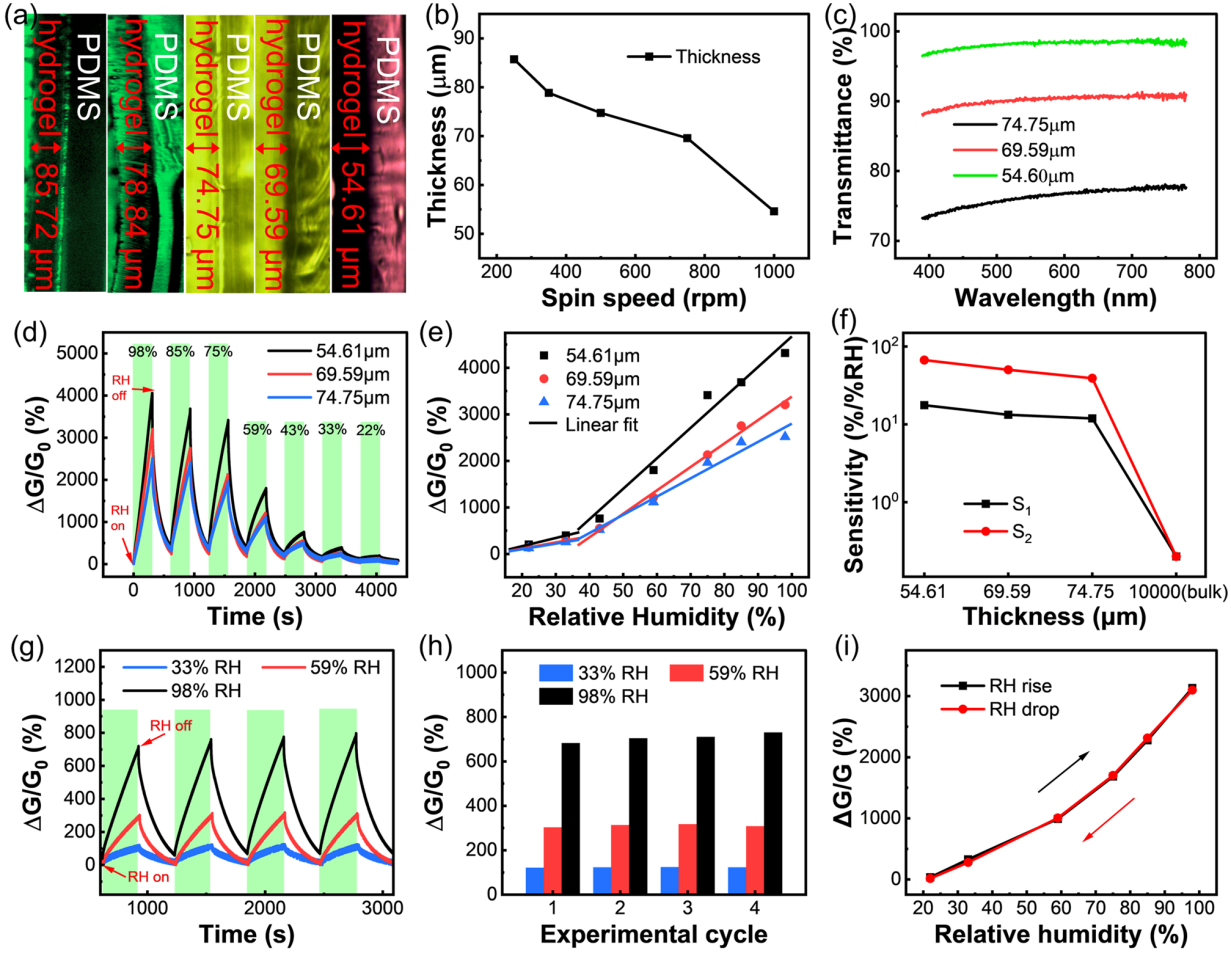
**a** Cross-sectional Optical microscope images showing the PDMS substrates and hydrogel films with different thicknesses obtained using different spin-coating speeds. **b** The correlation between spin coating speed and the thickness of hydrogel films. **c** Transmittance of film hydrogels with different thickness in the visible wavelength range. **d** Time-dependent responses of the humidity sensors with different film thickness upon exposure to different RH. **e** Piecewise fitting of the response of sensors with different film thicknesses versus RH. **f** The dependences of sensitivities (S_1_ under 11–37% RH and S_2_ under 37–98% RH) on the thickness of the film. **g** Time-dependent and repeated response of the sensor to 33, 59, and 98% RH in four experimental cycles, respectively. **h** Quantitative responses extracted from **g** versus experimental cycle for different RH. **i** Hysteresis curve of the sensor

Subsequently, we prepared a series of hydrogel films with different thicknesses by controlling the rotational speed of spin coating, and the effect of film thickness of hydrogel on the humidity sensing performance was then systematically investigated. By controlling the spin coating speeds at 250, 350, 500, 750, and 1000 rpm, the thicknesses of the obtained hydrogel films were 85.72, 78.84, 74.75, 69.59, and 54.61 μm, respectively, which were measured by microscope based on the cross-sectional profiles of the films (Fig. 4a-b). Notably, the thin-filmization can significantly increase the transparency of the hydrogel, and the transmittance gradually increases with decreasing thickness. This can be attributed to the weaker scattering effect of the hydrogel on incident light as the thickness decreases (Fig. 4c). As for the 54.61-μm-thick hydrogel film, the transmittance in the visible wavelength range is close to 100%, enabling the clear visualization of the object behind the device. Figure 4d shows the dynamic response curves of hydrogel films with different thicknesses to various humidity ranging from 98 to 22% RH, and their response versus RH curves are compared in Fig. 4e. The response of the hydrogel film humidity sensor increases with decreasing film thickness. It is because the specific surface area of the hydrogel increases rapidly owing to the thin-filmization, which accelerates the adsorption and desorption of water molecules by the hydrogel and then increases its humidity sensitivities (Fig. S4b). At the same time, its initial conductance decreases rapidly, which increases the response value of the sensor (Fig. S4a). Under the synergistic effect of the two, the humidity sensitivities (S_1_, S_2_) of the hydrogel sensor continued to increase significantly with decreasing thickness (Fig. 4f). As demonstrated in Fig. S7, the contact angle of water droplet on the film hydrogel tends to 0°. It means that the surface of the hydrogel film is super hydrophilic, which is extremely favorable to the adsorption of water molecules [59, 60], suggesting the great advantage of thin hydrogel films in humidity monitoring.

When used in practical applications, the repeatability of sensors is an important performance indicator that cannot be ignored [61–63]. As for the 54.61-μm-thick hydrogel film, we repeatedly exposed it to the same RH (33%, 59%, and 98% RH), and recorded its dynamic response curves, as shown in Fig. 4g. Figure 4h shows that the sensor has a response error within 6.6% to the same RH for 4 cycles, indicating its good repeatability as a high-performance humidity sensor. When the RH increased from 11 to 98% and then decreased to 11%, the response curves of the sensor were highly coincident, showing its small hysteresis (Figs. 4i and S8). Furthermore, the response and recovery time of the sensor are also evaluated, which are defined as the time required to attain a signal change of 90% of the full amplitude of response factor in one sensing cycle. Upon the change of RH from 11 to 98%, the response and recovery time of the hydrogel film-based sensor are measured as 275.6 and 227.0 s, respectively (Fig. S9a and Table 1). In actual breathing detection, the extreme RH variation will not occur. Whereas the sensor can respond to the RH changes caused by the respiration rapidly, showing the response/recovery time of 1.41/1.94 s, respectively, allowing for continuous breath monitoring (Fig. S9b-c).

**Table 1:** Comparison of the performances of humidity sensors based on various sensing materials

Sensing Materials	Sensitivity	Sensing Range (%RH)	Response/Recovery Time (s)/Humidity Change Range (%RH)	Flexibility	Transparency	Wireless Breath Monitoring
rGO/GO/rGO	45%/%RH	6.3–100	1.9/3.9 s/ 50^c^-100%RH	bendable	No	No [30]
Cleancool yarn	5,301%/%RH^a^	6–97	3.5/4 s/ 6–33%RH	bendable	No	No [31]
SWCNT/PVA	60%/%RH	60–100	200/340^a^ s/ 70–95%RH	150% tensile strain	No	No [32]
PDMS-CaCl_2_	3.13%/%RH^a^	30–95	60/42^a^ s/ 30–60%RH	20% tensile strain	No	No [33]
Poly-MMA/MAPTAC	0.67%/%RH^a^	20–90	35/310 s/ 10–85%RH	3% tensile strain	No	No [34]
CNF (with PEG)	1,680%/%RH^a^	20–90	265/490 s/ 20–90%RH	unstretchable	No	No [65]
TEMPO-oxidized cellulose paper	718%/%RH^a^	11–98	60/495 s/ 30–75% RH	bendable	No	Yes [66]
PAM/Tapioca	13,462.1%/%RH	11–98	276/227 s/ 11–98%RH	97% tensile strain	Yes	Yes^b^

^a^A value was not explicitly stated in the paper, but approximated from a graphical plot

^b^Present work

^c^Replace the room relative humidity in the article with 50%RH

**Fig. 5:**
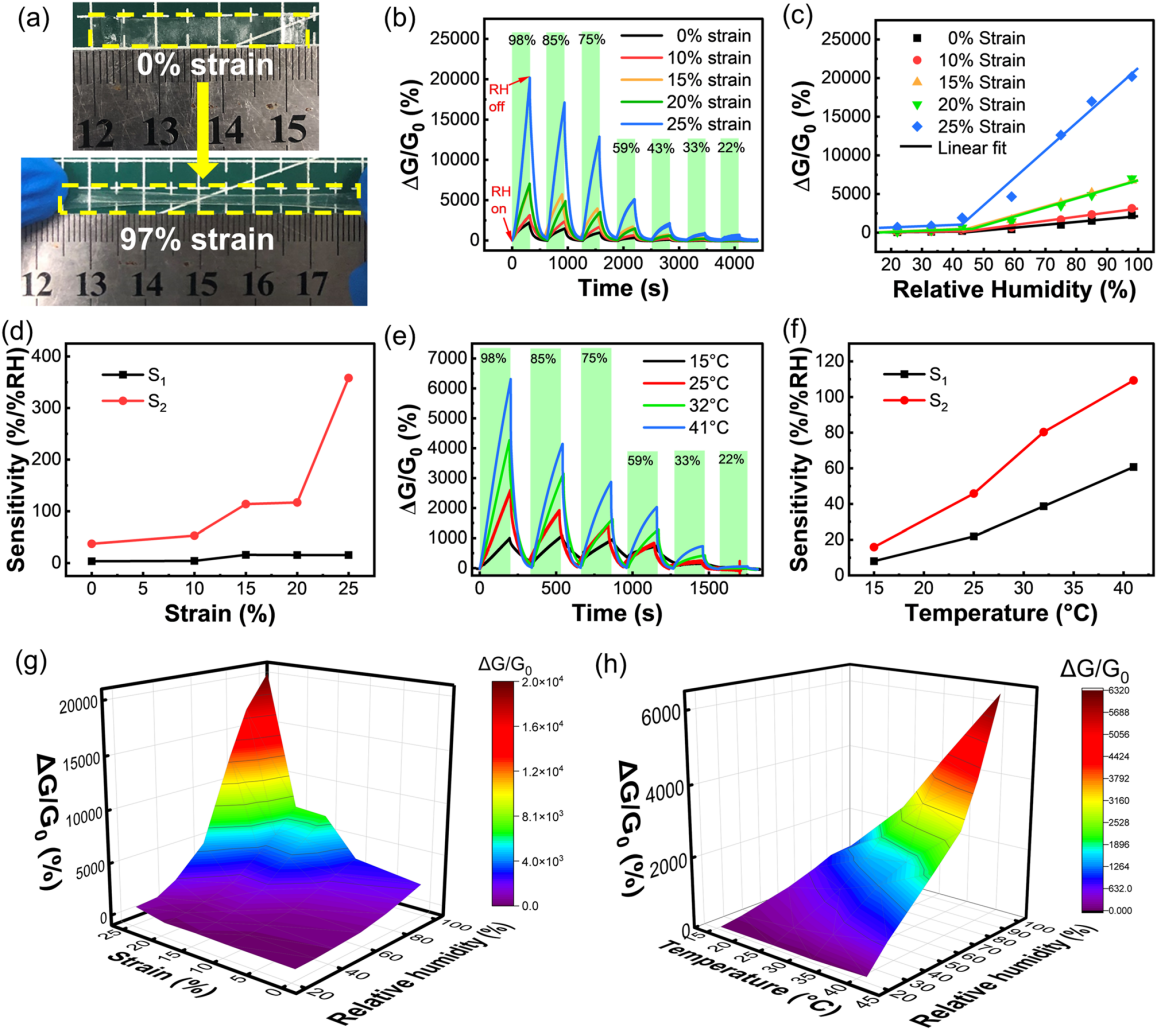
**a** Photographs show that the hydrogel film withstands 97% tensile strain. **b** Dynamic response curves to different RH at 0, 10, 15, 20, and 25% tensile strains. **c** Piecewise fitting of the response of sensors versus RH at different strains. **d** The relationship between sensitivity and tensile strain. **e** Dynamic response curves to different RH at 15, 25, 32, and 41 °C. **f** The relationship between sensitivity and temperature. **g** Compound plots of responses of the sensor toward strain and RH. **h** Compound plots of responses of the sensor toward temperature and RH

Compared with traditional humidity sensing materials, the superiority of the hydrogel is that highly stretchable humidity sensors that work normally under tensile strain can be fabricated based on it. Specifically, as shown in Fig. 5a, even the thinnest hydrogel film can withstand 97% tensile strain. To evaluate its humidity sensing performance under tensile strain, the 54.61-μm-thick hydrogel film was stretched to 10, 15, 20, and 25% strains, respectively, and then employed for humidity sensing (Fig. 5b). Attractively, the sensor exhibits higher responses to different RH after stretching (Fig. 5c), and the S_1_ and S_2_ of the sensor increased from 3.5 and 37.4%/%RH to 15.7 and 358.1%/%RH as the tensile strain gradually increases from 0 to 25% (Fig. 5d). Similar to the thin-filmization of the hydrogels, the stretching of hydrogel film can lead to the exposure of a larger specific surface area, and thus provide more adsorption sites for water molecules, thereby enhancing its sensitivity. This makes the humidity responding property of hydrogel mechanically programmable. In practical applications, a strain sensor can be integrated outside the hydrogel film humidity sensor to monitor the strain applied to the sensing system in real-time. The relative humidity in the environment can be accurately obtained by decoupling the humidity response from the compound signal by referring the compound plot (Fig. 5g). Therefore, by supplying a strain sensor as calibration in the future, this hydrogel film-based stretchable sensor can be used as a wearable device to accurately monitor humidity in practice with superior sensitivity.

Meanwhile, in daily use, the change of the external environment temperature may also affect the normal operation of the sensor. Therefore, it is necessary to investigate the influence of temperature on the humidity sensor. Humidity tests were performed by placing the sensor at 15, 25, 32, and 41 °C, respectively. The hydrogel film sensor can also work normally under daily temperature changes, and the higher the temperature, the greater response displayed by the sensor to the same RH (Fig. 5e-f). It is because the high temperature promotes the movement of water molecules, making the adsorption and desorption of the hydrogel film more efficient. To eliminate the interference of temperature on RH detection, a temperature sensor can be integrated outside the humidity sensor for calibration (Fig. 5h). This further demonstrates the feasibility of the hydrogel film humidity sensor to be used in daily life.

### Constituent Optimization for Further Improved Humidity Sensing

In addition to the design of microstructures, PAM-based hydrogels with better humidity sensing properties can be achieved through feasible materials engineering strategies. From this perspective, the free-standing PAM/tapioca DN hydrogel films were prepared in a similar way, where tapioca is a natural and green polysaccharide derived from the cassava root and can be regarded as a polycondensate of glucose. Especially, it is a good hygroscopic material and contains numerous hydroxyl groups that can bind with water molecules by forming hydrogen bonds, thereby greatly increasing the adsorption sites of water molecules in the hydrogel. Compared with the PAM/Carrageenan hydrogel film, the PAM/tapioca hydrogel film has not only the same high transparency (Fig. S10) but also certain self-adhesive properties due to the presence of more functional groups, this allows it to be directly adhered to human skin without the need for tape-like items (Fig. S11). Also, its mechanical properties are better than that of PAM/Carrageenan hydrogel films, alleviating the requirement of using PDMS as a substrate (free-standing hydrogel film) (Fig. S12), making the preparation process simpler and faster.

**Fig. 6:**
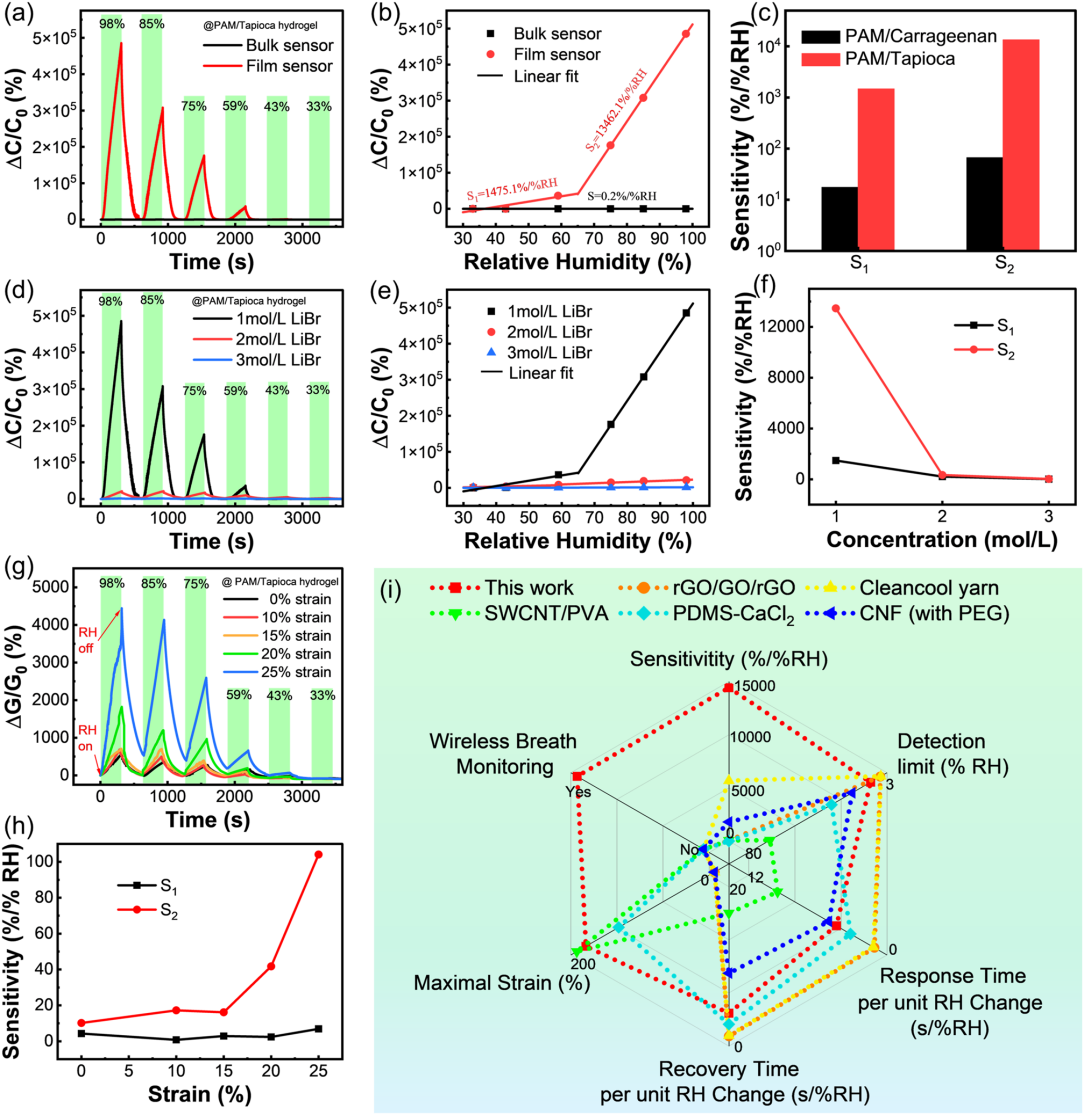
**a** Time-dependent responses (capacitance) and **b** piecewise fitting of the response of PAM/Tapioca DN hydrogel bulk and film sensors. **c** Sensitivity comparison of the two hydrogel sensors. **d** Dynamic response (capacitance) curves, **e** piecewise fitting of the response, and **f** sensitivity comparison of hydrogel film sensors soaked in 1, 2, and 3 mol L^−1^ LiBr solutions. **g** Dynamic response (conductance) curves and **h** sensitivity comparison of hydrogel film sensor at 0, 10, 15, 20, and 25% tensile strains. **i** The radar chart comparing the performance of this humidity sensor with that of others

Systematically, PAM/Tapioca hydrogel films with different thicknesses were also prepared separately by controlling the spin coating speed, and their humidity sensing performance was then tested in both conductance and capacitive modes. The capacitive mode provides another convenient and simple way to measure the humidity in many applications, and reflects the humidity-responsive behavior of hydrogel from another perspective. Similar to the conductance changes, the capacitance change of the hydrogel under different humidity is directly related to the reversible adsorption and desorption of water molecules. During this process, the relative permittivity of the hydrogel will change accordingly, and thus lead to the change of its own capacitance. In this case, the sensor response to RH is defined as *ΔC/C*_*0*_, where *ΔC* is the change in the capacitance of the sensor at the target RH relative to the initial conductance (*C*_*0*_) at 11% RH. For the bulk PAM/Tapioca hydrogel, the sensor exhibits only a small sensitivity of 0.2%/%RH. In stark contrast, the response of the PAM/Tapioca hydrogel film-based sensor is unprecedentedly boosted even compared to previous PAM/Carrageenan hydrogels (Fig. 6a-c), as a result of the synergistic modulation of structure and composition of hydrogel. For the thinnest PAM/Tapioca hydrogel film, its response reaches as high as 485,486.8% after exposure to 98% RH, and the sensitivity S_1_ in dry environment and S_2_ in humid environment increase to 1,475.1 and 13,462.1%/%RH, respectively (Fig. 6c), representing state-of-the-art performance standards. Like the PAM/Carrageenan hydrogel film, the PAM/Tapioca hydrogel film also displays remarkable thickness-dependent humidity responsiveness, demonstrating the universality of this thin-filmization method in improving the humidity transducing performance of different hydrogels (Fig. S13). Structurally, similar to the previous, the hydrogel film has a large specific surface area, which is conducive to the efficient adsorption of water molecules and leads to an explosion in response. Moreover, since this hydrogel film does not require PDMS as a supporting substrate layer, water molecules can be adsorbed simultaneously on both sides of the film without hindrance, leading to the improvement of the response due to the larger interaction surface area. Compositionally, apioca exhibits better hygroscopicity than carrageenan due to the exposure of more polar functional groups, providing significant advantages in humidity monitoring.

In addition, LiBr was introduced into the PAM/Tapioca hydrogel film in the form of Li^+^ and Br^−^, enabling good conductivity in a high-humidity environment. Also, there is a polarization between the two kinds of ions and water molecules, leading to a hydration reaction [64], which endows the hydrogel film with good environmental tolerance without the introduction of polyols. Considering the excellent hygroscopic ability of LiBr, we explored the effect of the concentration of soaked LiBr solution on the humidity sensing performance of PAM/Tapioca hydrogel films. As shown in Fig. 6d-e, the S_1_ of the hydrogel film humidity sensor soaked in 1, 2, and 3 mol L^−1^ of LiBr were 1,475.1, 210.3, and 13.5, respectively, and the S_2_ was 13,462.1, 334.2, and 25.6%/%RH, respectively. The response of the hydrogel film decreases significantly with the increase of LiBr concentration (Fig. 6f), which is mainly because the hydrogel film soaked with high concentration of LiBr has a large initial capacitance in dry environment due to the excellent hygroscopic ability of LiBr, resulting in a decrease in the response. Likewise, the response of the PAM/Tapioca hydrogel film to humidity in the conductance mode was also explored, including the effects of hydrogel structure, composition and LiBr concentration, and the results are presented in Fig. S14. Both the conductance and capacitance variations of the sensor exhibit similar trends due to the essential role of moisture adsorption and desorption during the humidity monitoring. Remarkably, this PAM/Tapioca hydrogel film-based sensor can also work under different tensile strains, and its response augments with increasing strain due to the decrease in film thickness, offering another route to optimize the sensing performance (Fig. 6g–h). Based on the above discussion, a stretchable hydrogel film-based sensor with predominant humidity responsiveness is developed through synergistic structural and compositional optimization, and this is undoubtedly a general strategy to fabricate a new generation of high-performance hydrogel-based humidity sensors. The overall performance of the hydrogel film-based humidity sensor is superior to that of existing flexible humidity sensor based on other transducing materials, such as rGO/GO/rGO, SWCNT/PVA and PDMS-CaCl_2_ (Table 1, Fig. 6i) [30–34, 65, 66]. Among them, the range of RH variation used for the calculation of response/recovery time is different in different articles. For a fairer comparison, we calculated the response/recovery time required for each 1% RH change for different sensors (Fig. 6i). Specifically, the hydrogel sensor displays much higher sensitivity, stretchability, transparency, and the ability to monitor respiration wirelessly when integrated in wearable mask.

### Proof-of-Concept Demonstration of Hydrogel Sensor for Respiratory Monitoring

**Fig. 7:**
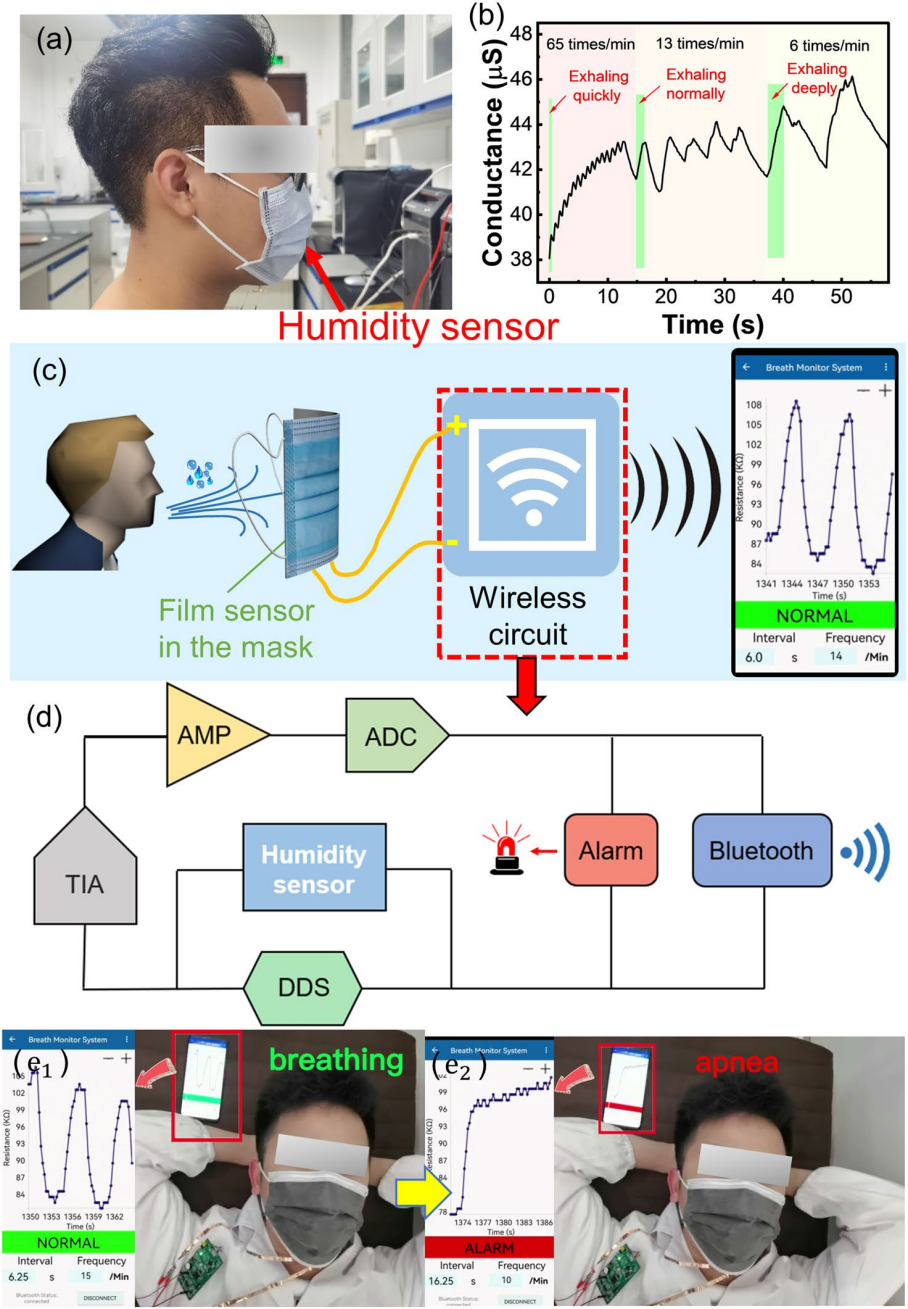
**a** Photograph showing the hydrogel film humidity sensor is integrated in the mask for real-time human respiration monitoring. **b** Dynamic response curves of the smart mask to fast, normal and deep breathing. **c** Schematic illustrating a wireless transmission system used to real-time monitor human breathing status and display the data on a mobile phone APP. **d** Schematic diagram of the principle of wireless circuit with Bluetooth transmission module and alarm. **e**_**1**_**-e**_**2**_ The APP displays "NORMAL" and "ALARM," respectively, during normal breathing and apnea

Respiration monitoring plays an important role in practice, in which the detection of humidity in exhaled air is an effective method to monitor respiration signals due to the large amount of water molecules contained in human exhaled air. Taking into account the superior humidity sensing capability, we explored the practical application of the flexible PAM/Tapioca hydrogel film-based humidity sensor for real-time respiration monitoring as wearable electronics. In detail, the sensor is closely attached to the inner side of the mask and utilized to monitor human respiration, as shown in Fig. 7a. When the human body exhales, the conductance of the sensor increases immediately due to the increasing RH around the nose; when the human body inhales, the conductance of the sensor decreases with the declining RH around the nose, and thus the final conductance curve appears with successive peaks during successive breaths. As a result, the frequency and depth of breathing can be obtained from the frequency and amplitude of these peaks, respectively. As shown in Fig. 7b, the hydrogel film humidity sensor can detect fast breaths of 65 times min^−1^, normal breaths of 13 times min^−1^, and deep breaths of 6 times min^−1^, respectively. The measured respiration frequency agrees well with the actual values.

Accordingly, we further integrate the sensor with a wireless circuit to construct a wireless sleep apnea detection and alarm system with Bluetooth transmission function, which can realize the acquisition, processing, transmission, and eventual display of the respiration state. The respiratory interruption alarm circuit provides a 600 mV, 200 Hz AC signal with a direct digital frequency synthesis (DDS) chip, and which is applied to the humidity sensor. The trans-impedance amplifier (TIA) converts and amplifies the current signal output by the sensor into a voltage signal, which is then collected by the analog-to-digital converter (ADC) of the STM32F103RCT6 after passing through a first-stage voltage amplifier. The STM32F103RCT6 is used as the Microcontroller Unit (MCU) to analyze the signal output by the humidity sensor, extract the waveform, frequency, and cycle of the respiration, and send the data to the mobile terminal in real-time through Bluetooth. At the same time, when the MCU detects that the breathing interval exceeds 10 s, the mobile phone and the circuit board trigger the sound and light alarms simultaneously to warn the user and the guardian to take action (Fig. 7c-d). As shown in Fig. 7e, when the user breathes normally, "NORMAL" sign was displayed in the middle of the interface of the APP, and the interval and frequency were also displayed at the bottom. As long as the sensor detects no breath within 10 s, "ALARM" sign was displayed in the APP and an alarm would be issued to warn people to take timely actions (Fig. 7e). The wearable and portable mask integrated with smart sensor can be widely used in daily life to prevent people from breathing interruption during sleep, which will endanger life and health. On the whole, this wearable humidity sensor based on hydrogel film has great potential to monitor human health status in a continuous, real-time and non-invasive manner.

## Conclusions

In summary, stretchable and transparent humidity sensors were constructed using PAM-based hydrogels as the transducing materials, boosting humidity sensing performance is achieved through synergistic regulation of structure and composition. On one hand, a general hydrogel film preparation method is proposed, and the resulting hydrogel films have thicknesses of tens of micrometers, high transparency in the visible light range, and more importantly, the boosted humidity sensing performance. This is attributed to the large specific surface area and small diffusion distance of water molecules in the hydrogel film, and the consequent more efficient adsorption and desorption of moisture. The improvement in humidity sensitivities brought by this method is much greater than that of ordinary micropatterns and wrinkled surfaces (Fig. S15), etc. The reduction in initial conductance also contributes to the increased sensitivities. On the other hand, the humidity sensitivity of the hydrogel films was further improved by introducing natural tapioca as the second cross-linked network instead. Thanks to the more polar hydrophilic groups and self-supporting double-sided exposed film structure, the free-standing PAM/Tapioca hydrogel film possesses excellent moisture adsorption capacity, and its sensitivity reaches unprecedented 13,462.1%/%RH.

Benefiting from the inherent stretchability of the sensing material, these humidity sensors are able to work properly under large tensile strains with even boosted sensitivity, which can meet the requirements of wearable devices. Also, the sensor exhibits excellent repeatability and broad detection range. By soaking the hydrogels with polyols or LiBr, their environmental tolerance is greatly improved, which is beneficial to the stability and reliability of the sensor. Notably, the in-depth humidity sensing mechanism of ion-conducting hydrogel is understood by the complex impedance spectrum. We have also attached the film humidity sensor to the inside of the mask to detect human breathing and uploaded the detected breathing interval and frequency to the APP on the mobile phone through the Bluetooth transmission module and specially designed circuit, which shows its great potential in wireless, real-time, continuous and accurate healthcare monitoring, such as the early detection and alarm of apnea in a facile way. This work provides a universal strategy to enhance the performance of hydrogel-based humidity sensors to adapt to practical wearable applications, which is of great significance for the development of advanced wearable electronics.

## Supplementary Information

Below is the link to the electronic supplementary material.

Supplementary file1 (MP4 63868 kb)

Supplementary file2 (PDF 1034 kb)
