# Supplementary material for: Humidity Sensing of Stretchable and Transparent Hydrogel Films for Wireless Respiration Monitoring
Source: Nanomicro Lett. 2022 Sep 12;14:183. doi: 10.1007/s40820-022-00934-1 (PMC9468213; doi:10.1007/s40820-022-00934-1)
Supplement: Supplementary file 2 — Supplementary file2 (PDF 1034 kb) [file 40820_2022_934_MOESM2_ESM.pdf]

Supporting Information for

Humidity Sensing of Stretchable and Transparent Hydrogel Films for Wireless Respiration Monitoring

Yuning Liang[1, #], Qiongling Ding[1, #], Hao Wang[1, #], Zixuan Wu[1], Jianye Li[1], Zhenyi Li[1], Kai Tao[2], Xuchun Gui[1], and Jin Wu[1, *]

[1]State Key Laboratory of Optoelectronic Materials and Technologies and the Guangdong Province Key Laboratory of Display Material and Technology, School of Electronics and Information Technology, Sun Yat-sen University, Guangzhou 510275, P. R. China

[2]Ministry of Education Key Laboratory of Micro and Nano Systems for Aerospace, Northwestern Polytechnical University, Xi'an 710072, P. R. China

[#]Yuning Liang, Qiongling Ding, and Hao Wang contributed equally to this work.

*Corresponding author. E-mail: wujin8@mail.sysu.edu.cn (Jin Wu)

Supplementary Figures

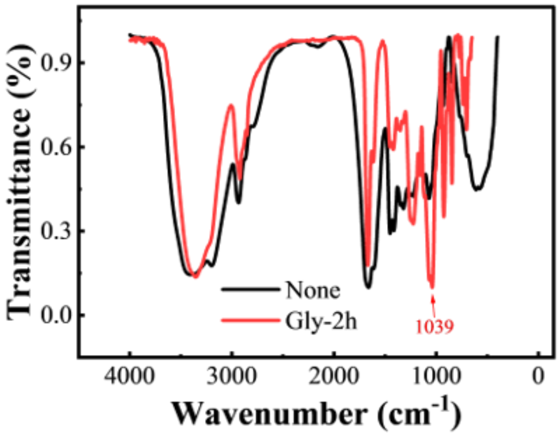

Fig. S1 FTIR spectra of PAM/Carrageenan DN hydrogels with (red) and without (black) soaking in glycerol for 2 h

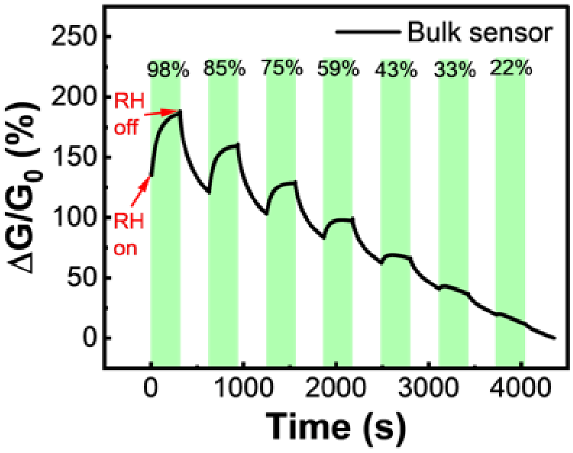

Fig. S2 Time-dependent responses of the bulk hydrogel sensor upon exposure to different relative humidity, presenting small response

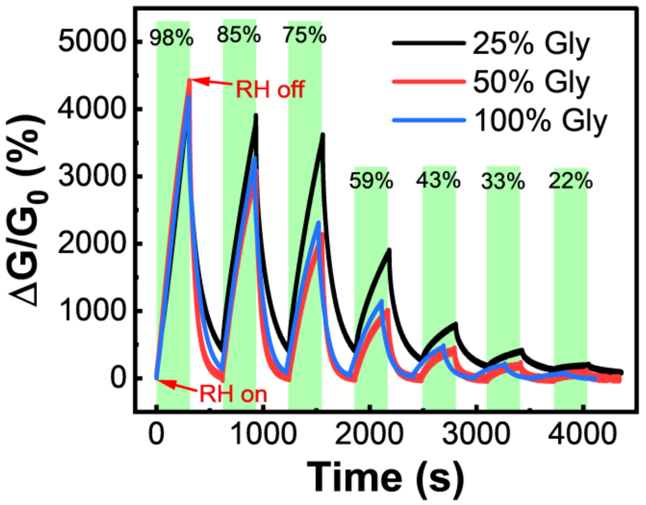

Fig. S3 Dynamic response curves of hydrogel film sensors soaked in 25%, 50%, and 100% Gly solution

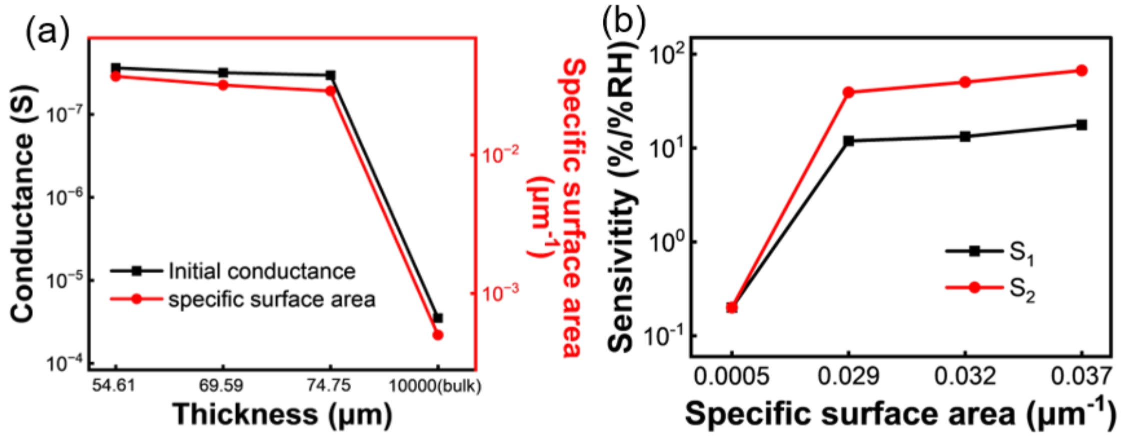

Fig. S4 **a** Relationship between hydrogel thickness and initial conductance and specific surface area of the sensor. **b** Relationship between specific surface area and humidity sensitivities of the sensor

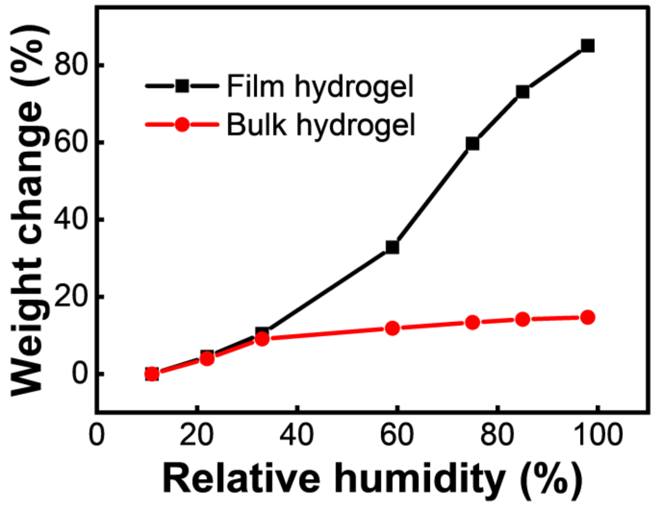

Fig. S5 Weight changes of film and bulk hydrogels versus the relative humidity. The weight changes are the weight variations of the samples relative to the initial weight at 11% RH after staying at different RH for half an hour

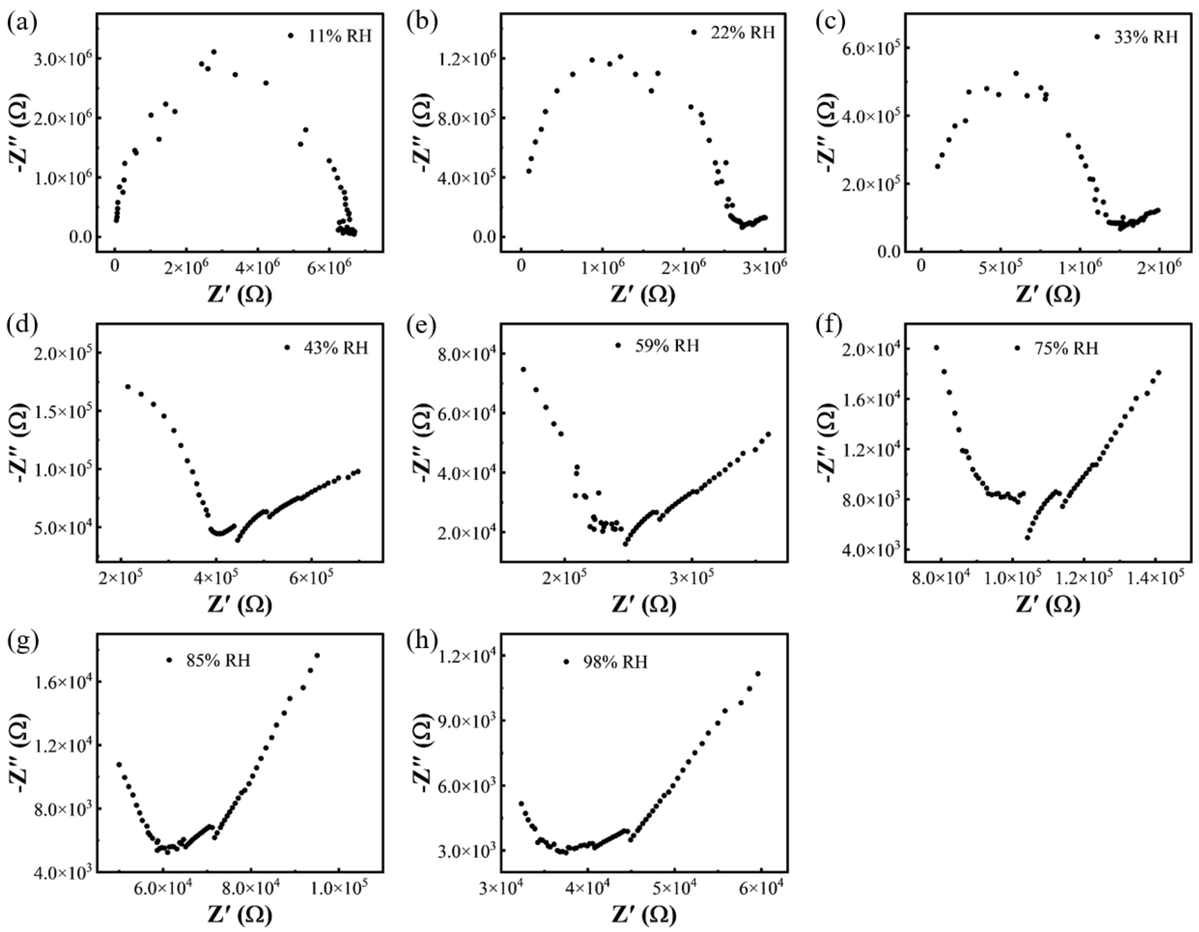

Fig. S6 a-h The complex impedance spectra of the PAM/Carrageenan hydrogel sensor under 11%, 22%, 33%, 43%, 59%, 75%, 85%, and 98% RH environment

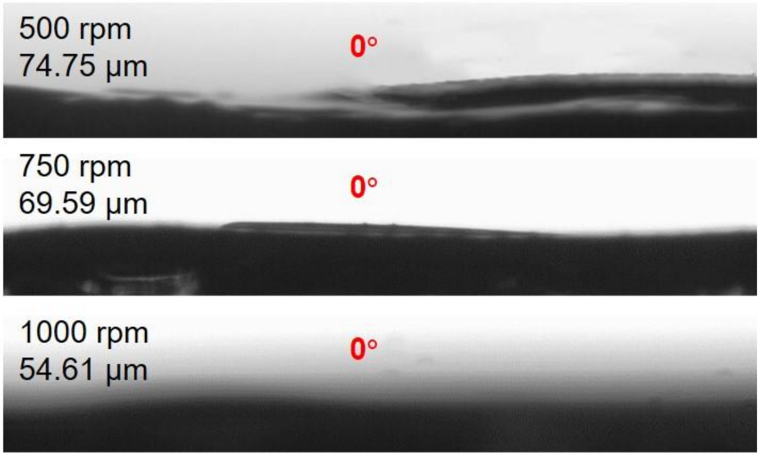

Fig. S7 Cross-sectional photographs showing the contact angles of water droplet on the hydrogel films with different thicknesses are 0°. The contact angles were measured using a contact angle measurement instrument (Beijing Audelino Instrument Co., Ltd. OCA15EC)

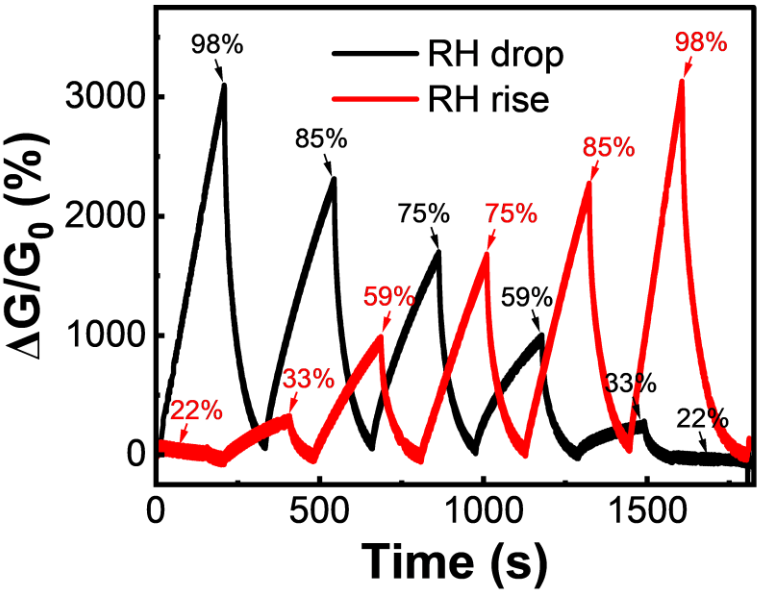

Fig. S8 Dynamic response curves of the humidity sensor with decreasing RH from 98% to 22% (black) and increasing RH from 22% to 98% (red), reflecting the small hysteresis

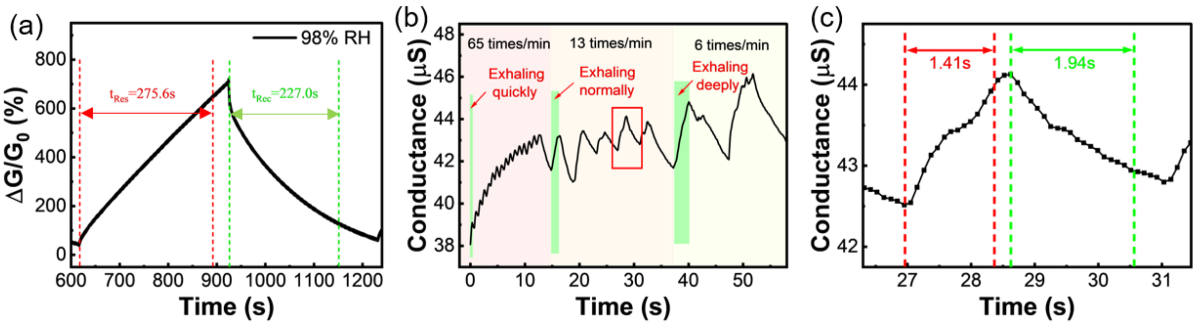

Fig. S9 a Analyses of the response and recovery time of the sensor in response to 98% RH. A cycle of normal respiration was taken from **b** (marked with red rectangle) as the research object to analyze the response time and recovery time of the sensor, which are 1.41 s and 1.94 s, respectively, as shown in **c**.

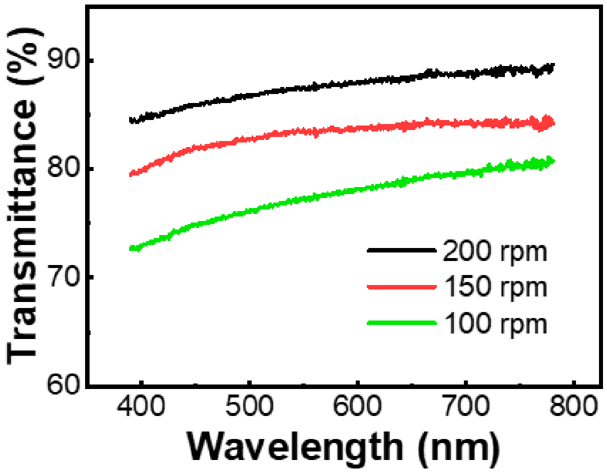

Fig. S10 Optical transmittance of PAM/Tapaoca film hydrogels with different thickness in the visible wavelength range

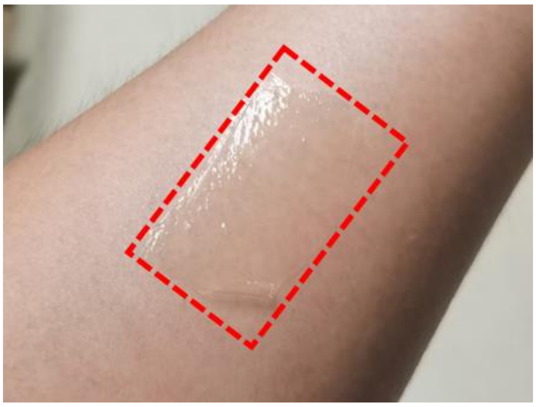

Fig. S11 Photograph showing the transparent and stretchable PAM/Tapioca DN hydrogel film is directly and conformally adhered to human skin

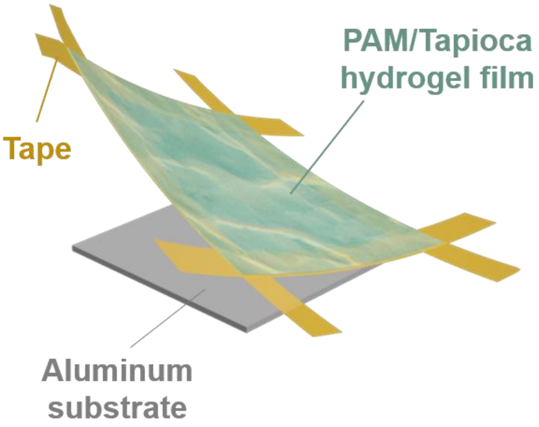

Fig. S12 Schematic diagram showing the preparation of free-standing PAM/Tapioca hydrogel film, which is utilized to fabricate stretchable humidity sensor

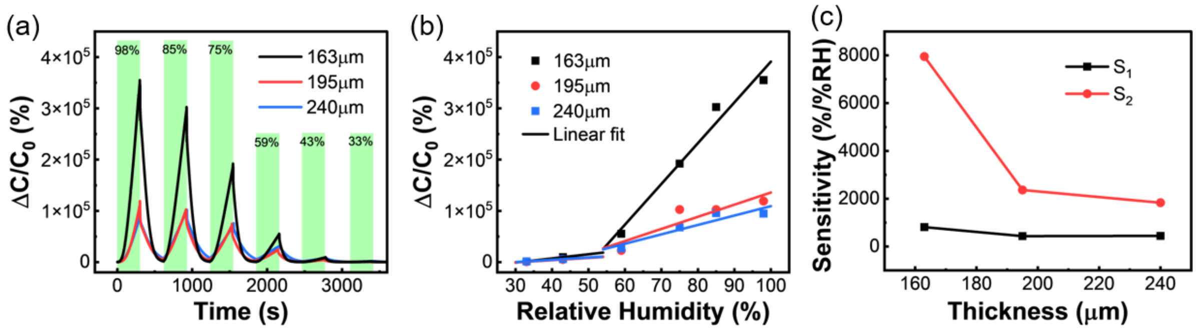

Fig. S13 Comparisons in **a** dynamic response curves, **b** piecewise fitting of the response, and **c** sensitivity of the PAM/Tapioca hydrogel film sensors with the thicknesses of 240, 195, and 163 μm in capacitance mode

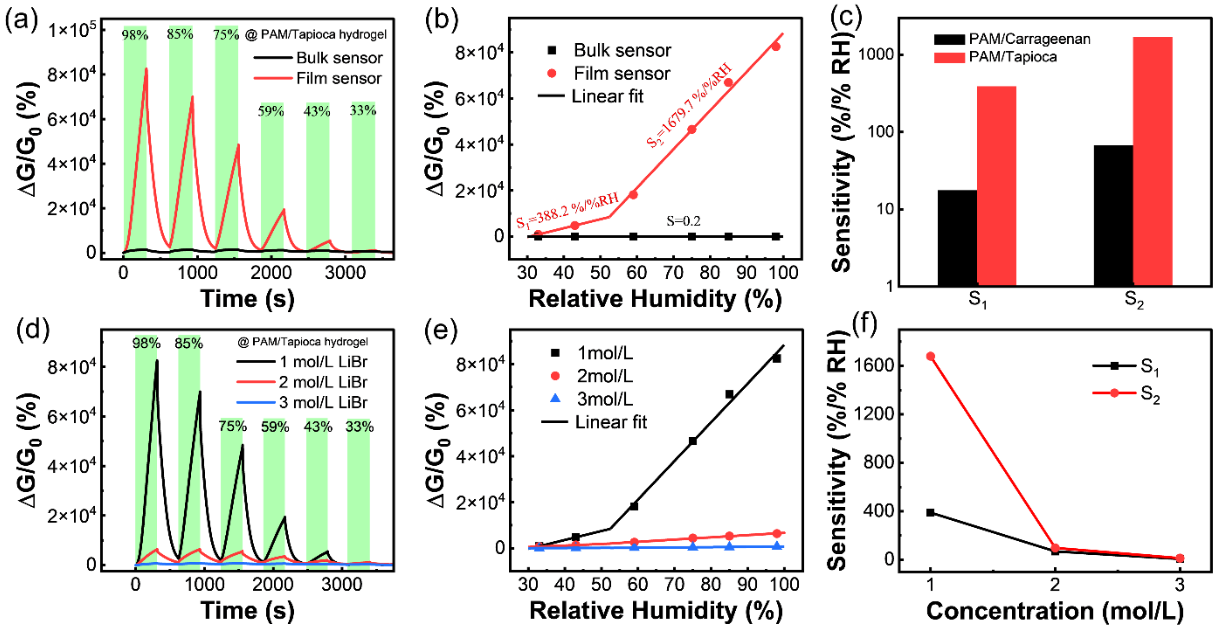

Fig. S14 a Time-dependent responses (conductance) and **b** piecewise fitting of the response of the PAM/Tapioca DN hydrogel bulk and film sensors. **c** Sensitivity comparison of two hydrogel sensors with different composition. **d** Dynamic response (conductance) curves, **e** piecewise fitting of the response, and **f** sensitivity comparison of hydrogel film sensors prepared with 1, 2, and 3 mol L^{-1} LiBr solutions

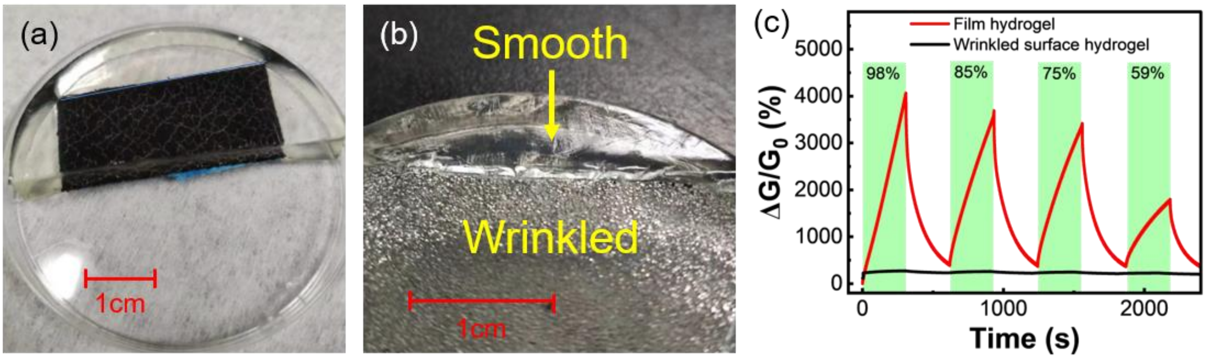

Fig. S15 a Coarse sandpaper with surface wrinkles is placed on the bottom of the petri dish, and then the precursor solution of the hydrogel is dropped on the surface for polymerization to obtain a hydrogel block with a wrinkled surface. **b** Picture of wrinkled-surface hydrogel vs smooth-surface hydrogel. **c** Dynamic response curves of film hydrogel and wrinkled-surface bulk hydrogel to different humidity. The responses of the film hydrogel sensor are much higher than that of wrinkled-surface bulk hydrogel for different RH
